# An observational study to test the acceptability and feasibility of using medical and nursing students to instruct clients in DMPA-SC self-injection at the community level in Kinshasa^☆^

**DOI:** 10.1016/j.contraception.2018.08.002

**Published:** 2018-11

**Authors:** Jane T. Bertrand, Dieudonné Bidashimwa, Paul Bakutuvwidi Makani, Julie H. Hernandez, Pierre Akilimali, Arsene Binanga

**Affiliations:** a Tulane School of Public Health and Tropical Medicine, Department of Global Health Management and Policy, 1440 Canal St, Suite 1900, New Orleans, LA 70112, USA; b Institut Supérieur de Statistiques, Kinshasa, Democratic Republic of Congo; c University of Kinshasa, School of Public Health, Kinshasa, Democratic Republic of Congo; d Tulane International LLC, Kinshasa, Democratic Republic of Congo

**Keywords:** Self-injection, Auto-injection, DMPA-SC, Injectable, Task-shifting

## Abstract

**Objectives:**

Given the promise of DMPA-SC to increase community-level access to modern contraception in developing countries, we conducted an observational study to assess the acceptability and feasibility of DMPA-SC self-injection among women in Kinshasa, Democratic Republic of the Congo, and of medical/nursing (M/N) students as instructors for self-injection.

**Study design:**

Women who selected DMPA-SC at a community outreach event adjacent to a health center were interviewed upon acceptance (baseline) and then 3, 6 and 12 months later.

**Results:**

Of 850 clients selecting DMPA-SC at baseline, 640 (75.3%) opted for self-injection over being injected by the M/N students for reasons of convenience and personal agency. Among these 640 self-injectors, 47.5% were anxious at baseline (for fear of needles or injecting incorrectly). Over 80% reported feeling very ready after training, confident that they knew how to self-inject and confident that they would remember the next injection date. By 3 months, 97% described it as easy. Half (54%) experienced side effects, mainly menstrual irregularities, the main reason for discontinuation. At 6-month follow-up, self-injectors cited effectiveness and ease of use as positive elements, though one quarter reported side effects. Their impressions of M/N students as instructors were highly positive.

**Conclusions:**

Where DMPA-SC was free and easily accessible, the majority of women interested in DMPA-SC opted to learn self-injection. The M/N students performed well in instructing women to self-inject. Clients were highly satisfied with the services received, yet many did not recognize their student status, possibly because outreach occurred near a health facility. Once told, clients remained very favorable, suggesting strong motivation to receive their preferred contraceptive free, whoever the provider.

**Implication statement:**

This study provides additional evidence on the acceptability and the feasibility of the self-injection of DMPA-SC by users from a resource-limited setting.

## 1 Introduction

The Democratic Republic of the Congo (DRC) has among the highest total fertility rates in the world (6.6 children) and a very low modern contraceptive prevalence rate (mCPR of 7.8% among women married or in union as of 2013–14) [1]. Recent research underscores numerous cultural, social and financial barriers to modern contraceptive use: fear of side effects (especially sterility), costs of the method, sociocultural norms (especially the dominant position of the male in family decision making), pressure from family members to avoid modern contraception and lack of information/misinformation [2].

Although societal norms reinforce large families, mCPR has increased in the past 5 years in the capital city of Kinshasa, from 18.5% in 2013 to 26.7% in 2017 [3]. As of 2017, injectable contraceptives represented 19.5% of modern method use among women in union [3]. In recent years, the DRC government has shown strong support for increasing mCPR [4], consistent with the National Multisectoral Plan for Family Planning: 2014–2020 [5]. This study tested an innovative strategy for increasing contraceptive access, which, if successful, could be scaled up to further contribute to the national effort of increasing mCPR.

DMPA-SC emerged in 2011 as a promising new option that could increase access to contraception, especially at the community level in low-income countries [6]. The product is a subcutaneous formulation of the intramuscular injectable contraceptive depot medroxyprogesterone acetate (DMPA-IM), available in the prefilled Uniject™ injection system [7]. Because it is effective, reversible and discrete, DMPA-SC has great potential to increase contraceptive use worldwide [8]. Further benefits include usability while breastfeeding, ease of administration and extremely low levels of unintended pregnancies [9]. Given its ease of administration, DMPA-SC lends itself to task-shifting to lower-level health care workers [7], [10] and to self-injection, which has yielded positive user experiences in seven countries where tested [9], [11], [12], [13] and increased continuation among users [14].

In the DRC, only physicians and nurses give injections. A 2015 pilot study in Kinshasa used medical and nursing (M/N) students to deliver DMPA-SC and other methods at the community level. It showed that women were highly satisfied with the method and service received from these providers [15], [16]. Local Ministry of Health (MOH) officials encouraged further testing of innovative strategies. The objectives of this study were to assess the feasibility of training M/N students to instruct women in the community to self-inject DMPA-SC; the willingness of clients to self-inject over receiving DMPA-SC from a provider; and user satisfaction with DMPA-SC as a method, self-injection as a procedure and counseling/instruction from the M/N students.

## 2 Materials and methods

### 2.1 Intervention

We carried out the study in three of the 35 health zones in Kinshasa: Kintambo (urban), Lingwala (urban) and Nsele (rural). The research team partnered with a local NGO, *Action en Santé et Dévéloppement* (ASD), which has extensive experience in family planning training, to oversee the implementation of the intervention. Specifically, ASD arranged for participation of one medical and five nursing schools, including supervisors and students from the 2015 pilot; obtained relevant health zone authorization; developed training curriculum for educating/coaching women to self-inject; and coordinated free family planning “campaign days” in the community with health zone personnel. The students received a vest with a family planning logo and knapsack containing contraceptives, supplies (foam cushion to practice self-injection, sterile gloves, lidocaine, alcohol) and data collection forms.

On campaign days previously announced to the community, 10–15 students arrived at a location adjacent to a health center and provided counseling and services (pills, condoms, CycleBeads or DMPA-SC) onsite to eligible clients. The students referred women to fixed facilities for methods requiring a trained family planning provider for initiation (e.g., IUD and implant). At baseline, women interested in DMPA-SC were encouraged to try self-injection, but they could instead choose the provider-administered injection.

Women opting for self-injection at baseline were invited to practice injection on a *mousse* (a thick piece of foam mimicking skin and approved as a mannequin by the DRC Ministry of Health); they then self-injected as the M/N student supervised. Students assessed competency to self-inject (on their own body) based on a checklist of 23 items that included Uniject preparation, skin disinfection, DMPA-SC injection and waste disposal, among others. Clients had to correctly perform 80% (> 18) of these items to be declared competent by the M/N students.

### 2.2 Data collection

Data collection for this research consisted of (a) surveys conducted by trained interviewers at baseline and follow-up at 3, 6 and 12 months among DMPA-SC clients; (b) a survey among the M/N students about their experience as providers; and (c) in-depth interviews with MOH and health zone personnel. This analysis is limited to the acceptor surveys (baseline and follow-ups). Interviewers entered data on Android smartphones that have been programmed with the Open Data Kit application; data were immediately transferred to a server, which the research team regularly monitored.

#### 2.2.1 Baseline

##### 2.2.1.1 Initial acceptor survey (Nov. 2016–Jan. 2017)

The intervention and data collection took place simultaneously in the three different health zones. Female interviewers experienced in contraceptive surveys received refresher training on the questionnaire content and survey procedures; they were present on campaign days. After a woman accepted DMPA-SC — either via self-injection or injected by the student — she was invited to participate in the baseline study. Clients who accepted the interview then moved from the student to the interviewer, who obtained informed consent. We aimed to enroll all DMPA-SC acceptors. However, because some acceptors were unable to wait while interviewers completed other interviews, we reverted to a convenience sample of clients available for the interview after receiving DMPA-SC. At baseline, interviewers obtained contact information for all participants willing to participate in follow-up surveys.

On all rounds of data collection, the wording on questions regarding the client's experience included four categories that the interviewer read: “very ___,” “somewhat___,” “not very ____” and “not at all” (anxious, satisfied, etc.).

##### 2.2.1.2 Three-month follow-up (Feb. 2017–May. 2017)

Community agents working for the health zone publicized a second round of campaign days, at which all DMPA-SC acceptors could receive a second dose. For clients who failed to return for the 3-month follow-up on campaign days, interviewers attempted to locate them in their homes or by phone. Because the research focused on DMPA-SC self-injection, only clients judged competent to self-inject by the 3-month follow-up were retained in the sample for the 6- and 12-month follow-up. At 3 months, these clients received three doses of DMPA-SC to cover home self-injection at 6, 9 and 12 months.

##### 2.2.1.3 Follow-up at 6 months (May–Jul. 2017) and 12 months (Nov, 2017–Jan. 2018)

**Fig. 1:**
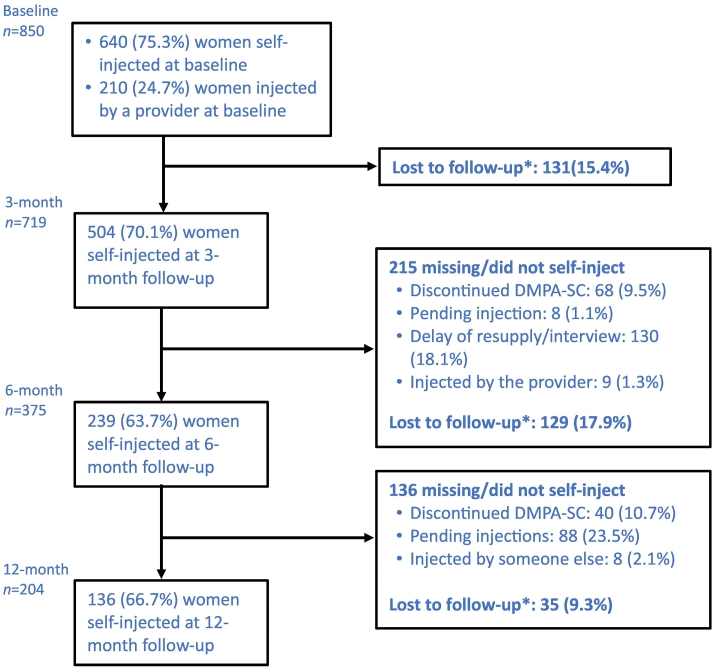
Participant flow diagram for the DMPA-SC self-injection study in Kinshasa, DRC. *The sample size (*n*) on the previous round is used as the denominator for the subsequent round.

After the first two rounds, there were no further campaign days. The research team located self-injectors (1) by phone, to establish a time and place for the interview, or (2) in their homes, via the community extension worker. Fig. 1 presents the number of cases retained at each round and the reasons for attrition. Over different rounds, some questions were dropped and new ones added to track self-injectors' experience over time.

### 2.3 Analysis

The researchers used Stata (version 13) to complete a descriptive analysis of the data.

To assess a possible selection bias resulting from attrition, we treated each survey (baseline, 3-month, 6-month and 12-month) as independent from each other; then, we conducted bivariate analyses, *χ*^2^ statistics for categorical variables and analysis of variance (ANOVA) for continuous variables to compare the baseline sample to each follow-up sample on sociodemographic characteristics. We assessed statistical differences at an alpha level of .05.

Although this approach might not be best suited for longitudinal designs and could potentially lead to a lower likelihood to find significant differences, it was the best available approach given that we were unable to track participants as a unique longitudinal sample.

This research received human subjects approval from Tulane University (#911338-7) and the Kinshasa School of Public Health (#ESP-CE/071/2016). All the participants provided written consent before their inclusion in this research.

## 3 Results

### 3.1 Willingness to self-inject among women selecting DMPA-SC on campaign days

On the first campaign days held for the purposes of this study, a total of 850 women chose DMPA-SC as their method. Of these, 640 women (75.3%) opted for self-injection over an injection by the M/N student. After being trained, the clients self-injected in front of the student. Women choosing not to self-inject cited fear of injecting incorrectly, fear of hurting themselves and greater trust in the provider (data not shown).

### 3.2 Sociodemographic characteristics of self-injectors at baseline and follow-up surveys

At baseline, the 640 DMPA-SC self-injectors were 27 years old on average; only one in five had completed secondary school. Three quarters were married or in union; close to 100% had at least one child, with the average being three; two thirds wished to have more children, often after several years. Only half were employed (and of those, most were paid in kind, not cash) (Table 1).

**Table 1:** Sociodemographic characteristics of women opting for DMPA-SC self-injection through a community-level program in Kinshasa, DRC

Sociodemographic characteristics	Baseline (*n*=640)	3-month follow-up (*n*=504)	6-month follow-up (*n*=239)	12-month follow-up (*n*=136)
Age in years				
Mean	26.7	27.4	28.5***	28.5**
(SD)	(6.5)	(6.8)	(7.1)	(6.9)
Last year, education				
Primary or no education	21.1	17.3*	14.6	15.4
Some level secondary	56.9	64.1	59.8	56.6
Completed secondary or higher	22.0	18.7	25.5	27.9
Married or in union	75.9	73.2	71.1	69.1
Has living children	99.1	98.4	99.6	98.5
Number of children				
Mean	3.1	3.2	3.4*	3.5*
(SD)	(1.8)	(1.9)	(2.1)	(1.8)
Employed	50.6	44.4*	44.4	47.1

The asterisks denote a statistically significant difference between the value obtained on this round and the baseline based on ANOVA and *χ*^2^ tests on the samples in baseline and three follow-up surveys: *≤.05, **≤.01, ***≤.001 (exact values available on request).

This study was conducted in a community context, without the benefit of client records available through fixed health facilities. We experienced severe loss to follow-up between surveys. Due to logistical problems, the student providers and interviewers returned 1 week late for the designated 3-month follow-up/campaign days (potentially affecting an estimated 15% of DMPA-SC acceptors interviewed at baseline). Interviewers were present on the designated campaign days for the rest of the 3-month follow-up (and all subsequent data collection). They interviewed all baseline respondents (provider-injected or self-injected DMPA acceptors) who attended, as well as attempting to locate others by phone or home visit.

At 3-month follow-up, 131 cases (15.4%) were lost to follow-up. Among the 719 located, 504 (70.1%) self-injected at 3 months; 68 (9.5%) reported discontinuing DMPA-SC; eight (1.1%) had a pending injection; 130 (18.1%) were interviewed but dropped from the analysis because of the above-mentioned delay of resupply/interview at 3 months; and nine (1.3%) requested injection by the provider. At 6 months, an additional 129 (17.9% of 3-month sample) were lost to follow-up. Among the 375 clients located, 239 (63.7%) reported to have self-injected at 6 months, 40 (10.7%) had discontinued DMPA-SC, eight (2.1%) received the injection from someone else, and 88 (23.5%) still expected to self-inject. By 12 months, we located 204 clients; 136 (66.7%) reported to have self-injected at 12 months, whereas 24 (11.8%) had discontinued DMPA-SC, three (1.5%) received the injection from someone else, and 41 (20.1%) still expected to self-inject; see Fig. 1. In short, 75.3% of the 850 DMPA-SC clients at baseline opted for self-injection. The percentage of the original 850 whom we located, interviewed and determined to still be self-injecting was 59.3% (at 3 months), 28.1% (6 months) and 16.0% (12 months). These percentages should not be confused with continuation rates due to the large loss to follow-up. Over the course of the research, respondents in later rounds of the survey tended to be slightly better educated but less likely to be married or employed.

### 3.3 Contraceptive history among self-injectors at baseline (*n*=640)

Less than half of the self-injectors (46.3%) had ever used a contraceptive method; 15.8% had ever used an injectable, and 2.2% had used DMPA-SC (Table 2). Among previous injectable users, most (61.4%) used an appointment card to remember the date of the next injection, with far fewer mentioning a calendar, note to self or “just remembered.” Despite these aids, 28.7% had missed an injection. Most previous injectable users (77.2%) had stopped using the injection because of desire to get pregnant, concern about (future) fertility, nonavailability of the method, side effects or a health problem. Among previous users of any method, half had used male condoms and withdrawal, followed by rhythm and the pill.

**Table 2:** Baseline contraceptive history of users of DMPA-SC self-injection in Kinshasa, DRC

Variables	Baseline (*n*=640)
Previous users of any contraceptive method	46.3
Previous users of an injectable	15.8
Previous users of DMPA-SC	2.2
Among previous users of an injectable:	*n*=101
Strategies to remember reinjection date:	
Appointment card	61.4
Calendar	22.8
Note	15.8
Memorization	15.8
Other	1.0
Ever missed an injection	28.7
Stopped using an injectable	77.2
Reasons for stopping an injectable:	
Wanted another pregnancy	39.7
Concerns about fertility	16.7
Nonavailability of method	14.1
Side effects	11.5
Other health problems	11.5
Contraceptive use after stopping the injectable changed method:	30.8
Stopped using contraception	66.7
Can't remember	2.6
Among previous users of any method;	*n*=195
Contraceptive methods ever used:	
Male condom	54.4
Withdrawal	52.3
Calendar	43.6
Pill	27.2
Other	21.0
Most recent method used:	
Withdrawal	28.7
Male condom	23.1
Rhythm	18.0
Pill	16.4
Other	13.9

For 61.7% of the self-injectors, DMPA-SC was their method of choice; almost all the remainder would have preferred an implant. Half (48.2%) came for services based on their own decision; for 37.8%, it was a joint decision with their husband or partner. Among those married or in union, about three quarters reported that their husband or partner was favorable to family planning. However, at least a quarter felt that some members of their community, including family and friends, opposed family planning (data not shown in tables).

### 3.4 Experiences of DMPA-SC self-injectors at baseline and follow-up at 3, 6 and 12 months

At baseline (*n*=640), most acceptors chose self-injection because it would be easy to perform at home (58.8%); one in five also mentioned liking to learn new things and to be able to manage this product themselves. Four in five considered themselves to be “very motivated” to try self-injection. Almost half (47.5%) reported being somewhat or very anxious at their first injection because of fear of the needle or pain. Close to 30% reported pain during the first injection, but less than 10% felt pain afterwards (data not shown.)

At baseline, 65.2% reported the first self-injection was somewhat or very easy (those finding it difficult mentioned problems of inserting the needle and pumping the medication). Most (90.3%) felt very well prepared by the training from the M/N students to perform self-injection. Over 90% felt confident to perform self-injection, to follow the instructions in the booklet on self-injection and to remember the date for their next injection (Table 3). Most (67.5%) planned to rely on an appointment card (*jeton)* to remember the date of the next injection, with far fewer mentioning the calendar, a written note or “just remembering.” Only 8.0% expressed that they would want to seek help from a family member or someone else for the next injection.

**Table 3:** Experience of the DMPA-SC self-injectors in Kinshasa, DRC, at baseline and at 3-, 6- and 12-month follow-up surveys

Variables	Baseline^a^ (*n*=640)	3-month follow-up (*n*=504)	6-month follow-up (*n*=239)	12-month follow-up (*n*=136)
Level of anxiety about self-injection compared to previous round				
As anxious	-	5.6	10.0	11.8
Less anxious	-	86.9	84.9	80.2
More anxious	-	7.5	3.4	4.4
No answer	-	0.0	1.7	3.7
Level of difficulty of performing self-injection				
Very difficult	14.8	2.8	-	-
Somewhat difficult	20.0	5.6	-	-
Somewhat easy	24.7	22.6	-	-
Easy	40.5	68.9	-	-
Level of difficulty of the most recent self-injection compared to the previous round		*n*=399		
Less difficult	-	90.5	73.2	86.5
As difficult	-	9.0	23.9	12.0
More difficult	-	0.5	2.9	1.5
Reason of difficulty about self-injection (multiple answers allowed):	*n*=223	*n*=43		
Inserting the needle	75.3	67.4	-	28.6
Express all the liquid	27.4	20.9	-	25.0
Knowing where to inject	14.8	-	-	-
Everything was easy	11.7	-	-	-
Remembering the date	-	-	-	14.3
Prepare DMPA-SC	-	27.9	-	17.9
Prepare skin	-	14.0	-	-
Waste disposal	-	-	-	28.6
Other	13.9	14.0	-	-
Strategies to remember the date of next injection (multiple answers allowed):				
Appointment card/jeton	67.5	-	68.2	54.4
Calendar	12.7	-	5.0	7.4
Note	11.3	-	-	-
Just remember	7.2	-	27.6	44.1
Other	1.4	-	9.2	8.8
Positive aspects of using DMPA-SC in self-injection (multiple answers allowed):				
Effective to prevent pregnancies	-	-	66.5	65.4
Easy to use	-	-	45.2	61.0
Allows to control fertility decisions	-	-	20.1	8.8
Less side effects	-	-	13.4	7.4
Easy to hide	-	-	-	16.9
Protects for a long time	-	-	-	14.7
Other	-	-	31.0	5.9
Negative aspects of using SMPA-SC in self-injection:				
No negative aspects	-	-	43.1	61.8
Side effects	-	-	25.5	21.3
No answer	-	-	19.7	7.4
Doses are hard to obtain	-	-	5.4	6.6
Other	-	-	7.1	1.5
Level of confidence about how to perform self-injection of DMPA-SC:				
Very confident	83.59	-	-	-
Confident	12.34	-	-	-
Somewhat confident	1.88	-	-	-
Not very confident	0.0	-	-	-
Not confident	2.19	-	-	-
Level of confidence the self-injection of DMPA-SC was performed correctly:				
Very confident	-	89.1	-	-
Confident	-	0.0	-	-
Somewhat confident	-	9.1	-	-
Not very confident	-	0.8	-	-
Not confident	-	1.0	-	-
Level of confidence about following the instructions in the booklet to self-inject DMPA-SC:				
Very confident	80.16	-	-	-
Confident	12.97	-	-	-
Somewhat confident	0.78	-	-	-
Not confident	2.34	-	-	-
Don't know	3.75	-	-	-
Level of confidence about when to perform to perform the injection:				
Very confident	83.44	94.4	-	-
Confident	13.59	-	-	-
Somewhat confident	0.78	4.2	-	-
Not confident	1.88	0.5	-	-
Don't know	0.31	0.8	-	-
Willing to seek help from family member or friends for the next injection:	8.0	-	0.0	1.5
To whom will you ask for help?				
Community-based distributor	70.59	-	-	-
Health care provider	31.37	-	-	75.0
Friend	13.73	-	-	-
Other family member	13.73	-	-	-
Other	7.84	-	-	25.0

a All percentages are based on the *n* of 640, 504, 239 and 136, respectively, for baseline and 3-, 6- and 12-month follow-up except where indicated to the contrary.

At 3 months (*n*=504), the experience with self-injection was similar, though 86.9% mentioned feeling less anxious and 90.5% found it less difficult than at baseline. Nine in 10 (89.1%) felt very confident that they had self-injected correctly. Half (54.1%) reported side effects, the most common being irregular period, heavy and frequent bleeding, no period and abdominal pain. Some 20.4% had experienced skin reactions at the site of injection after self-injection, but among these women, only 1.9% had sought treatment for the reaction (Table 4).

**Table 4:** Safety of DMPA-SC self-injection among users in Kinshasa, DRC, at 3-, 6- and 12-month follow-up surveys

Variables	3-month follow-up *n*=504	6-month follow-up *n*=239	12-month follow-up *n*=136
Skin reactions after self-injection	20.4	12.5	23.5
Sought help for skin reaction	1.9	0.0	3.1
Ever experienced side effects while using DMPA-SC	51.4	-	58.8
Side effects ever experienced			
Heavy bleeding	38.6	-	8.8
No period	33.6	-	61.3
Irregular period	31.3	-	28.8
Weight gain	-	-	8.8
Other	13.5	-	7.5
Relative severity of side effects (percentage of users reporting [side effect] was tolerable or somewhat tolerable):			
Irregular periods	-	-	95.7
No period	-	-	83.7
Heavy bleeding	-	-	85.7
Weight gain	-	-	100.0
Help seeking for the side effects:			
Irregular period	-	-	30.4
No period	-	-	22.5
Heavy bleeding	-	-	71.4
Weight gain	-	-	0.0
Evolution of side effects (percentage of users reporting [side effect] had resolved by the time of the interview):			
Irregular period	-	-	39.1
No period	-	-	16.3
Heavy bleeding	-	-	57.1
Weight gain	-	-	14.3

Confidence remained high that they would remember the date of the next injection (98.7%) and that they would correctly perform it next time (99.5%).

At 6-month follow-up (*n*=239), respondents cited the effectiveness of DMPA-SC in preventing pregnancies (66.5%) and its ease of use (45.2%) as the most positive aspects of DMPA-SC self-injection. The negative aspects of self-injecting DMPA-SC included its side effects (25.5%) and unavailability of the method (5.4%); however, 43.1% reported no negative aspects of self-injecting DMPA-SC (Table 3).

The findings from the 12-month follow-up (*n*=136) produced few new results compared to the 6-month follow-up; 13.5% still found self-injection “as or more difficult” as when they started, with inserting the needle and expressing the medication still the main reasons. By contrast, 61.8% reported no negative aspects to self-injection.

Almost all self-injectors at 3, 6 and 12 months expected to self-inject in the future. Reasons given (at 3-month follow-up) were their confidence that they could do self-injection and the convenience of not having to go to the health facility.

### 3.5 Reasons for discontinuation

Given the large loss to follow-up between rounds of data collection, we have not presented data on discontinuation rates. However, of 76 self-injectors who discontinued between baseline and 3 months, the main reasons were the desire to stop the method (*n*=45), partner opposed (*n*=12) and change to another contraceptive method (*n*=11). At 6- and 12-month follow-up, discontinuers frequently cited fear of side effects (data not shown).

### 3.6 Management of doses of DMPA-SC at home (6-month follow-up; *n*=239)

Clients judged competent to self-inject at 3 months were to receive three doses of DMPA-SC for subsequent reinjections at 6, 9 and 12 months at home. Most self-injectors (80.5%) reported that 3 months earlier, they had received at least one dose of DMPA-SC to take home (one dose: 2.9%, two doses: 22.2%, three doses: 76.0%). Almost all had a safe place to store it at home (e.g., in a closet, suitcase, handbag). Most disposed of the waste in trashcans (51.5%), latrines (42.3%) or “discarded outside” (11.6%) (data not shown.)

### 3.7 Satisfaction with the M/N students as educators in self-injection at baseline (*n*=640)

This study also assessed client satisfaction at baseline with the performance of the M/N students in teaching women to self-inject. Four in five (80.5%) of the initial self-injectors did not realize that the provider was a student. Yet most were very (78.8%) or somewhat (9.7%) comfortable about learning to self-inject from a student. Most felt the M/N students were comfortable in explaining the method and its side effects (94.4%), and how to self-inject (99.4%). Close to 90% of acceptors were very satisfied with the information and counseling they received; 95.9% of acceptors would “strongly recommend” or “somewhat recommend” DMPA-SC self-injection to others.

At the 3-month follow-up, the findings were similar: 95.6% felt that the M/N students were very knowledgeable; 87.9% considered the explanations to be clear; and 93.5% found the M/N students to be “very respectful” toward them.

### 3.8 Feasibility of using M/N students to instruct clients in DMPA-SC self-injection

“Feasibility” was not measured by a specific set of variables in a survey but rather by demonstration of the following:

1. Is it possible for the D6 to train M/N students in family planning as part of their nursing curriculum?
2. Once trained, are M/N students capable of instructing clients to self-inject DMPA-SC at the community level?
3. Is the use of nursing students to deliver contraception in the community compatible with the existing system for delivering primary health care at the health zone level?

The experience of this research pilot provided strong evidence that all three are possible, further strengthening support for institutionalizing the family planning curriculum in the country's nursing schools.

## 4 Discussion

This study demonstrated the feasibility of training M/N students to teach women at the community level to self-inject DMPA-SC in Kinshasa, DRC. It also showed that three in four clients interested in DMPA-SC were willing to try self-injection, at least in a setting where contraception was free and easily accessible.

Clients reported high levels of satisfaction both with DMPA-SC as a method and with self-injection as a procedure. Despite initial anxiety, by 3 months, they gained a high level of confidence in their ability to self-inject; few reported difficulties with the procedure or pain from it. Also, clients gave positive feedback regarding the performance of the M/N students: they were knowledgeable about DMPA-SC, they gave clear explanations, and they were respectful of the clients.

Our findings on the acceptability of DMPA-SC as a method are consistent with the results of the pilot introduction of the method in Burkina Faso, Niger, Senegal and Uganda from 2014 to 2016 [17], as well as our 2015 pilot in Kinshasa [16]. Our findings on high client satisfaction with self-injection concur with results from studies in Malawi and Uganda [18], [19]; clients who self-injected had higher continuation rates than those who received DMPA-SC from providers. In Niger, Senegal, Malawi and our studies in Kinshasa, the providers were community-based agents. By contrast, in the studies from Burkina Faso and Uganda, providers were clinic-based (e.g., trained nurses).

An important limitation of the research was the high loss to follow-up of DMPA-SC acceptors interviewed at baseline, resulting from (1) the team's delayed return for the 3-month follow-up (potentially affecting 15% of the baseline sample) and (2) difficulty in re-locating clients in communities where street names are not posted and houses often than have no numbers. Although clients could refuse the follow-up interview (when asked at baseline), some may have given false phone numbers to avoid further contact, especially if members of their household did not know they were using contraception.

This observational study was conducted under real-life conditions in highly impoverished neighborhoods of Kinshasa, with the aim of approximating the likely conditions in future expansion of this approach. In contrast to other self-injection studies where clients were recruited and taught to self-inject in a clinical setting [20], this study recruited clients through the mechanism of campaign days. Although the location for the campaign day was often adjacent to a health center, the project staff did not attempt to register the names of women in a client base, nor did they establish individual client records, as would be done in a clinic setting. For this reason, we do not report the proportion of acceptors enrolled in the study. Although less controlled, the “campaign approach” allowed women to obtain family planning counseling and the method of their choice (among the four available from the M/N, students) for free, with relatively little waiting time. By contrast, local health facilities charge for clinic inscription as well as the cost of the method, which often makes contraception unaffordable to this population. In addition, women often have to wait for 3–4 h or longer to receive family planning services in a clinic setting. However, the lack of detailed information on the clients served, including individual client records, hindered efforts to re-locate self-injection acceptors for the follow-up surveys. In short, the campaign approach increased access to modern contraception for women in these impoverished communities, yet it contributed to high loss to follow-up.

The implications of this high loss to follow-up over the course of the study are unclear. Data in Table 1 show that self-injectors followed through 6 and 12 months were slightly older and had more children. One hypothesis is that age might contribute to better adherence to a method, whereas having more children might provide additional motivation to use contraception. However, the magnitude of difference is small, suggesting a similar sociodemographic profile across the four rounds of data collection, despite attrition.

Although not reported herein, results from in-depth interviews with MOH officials and health zone officials indicated widespread acceptance for DMPA-SC self-injection as a method and for the use of M/N students to motivate and instruct women in its use in the community. The research team disseminated results from the studies of acceptors, M/N students and health authorities in Kinshasa in November 2017 as a first step to authorization for scale-up of this approach. In September 2018, the Minister of Health endorsed the scale-up of this approach, in addition to two other community-level strategies [22].

Efforts are already under way to institutionalize the use of M/N students for community-based distribution of contraceptives, including both DMPA-SC and Implanon NXT through the *6ème Direction* of the MOH, which is responsible for the network of nursing schools throughout the DRC.

Whereas the pilot research provides a clear model for operationalizing the use of nursing students to administer contraception, the scale-up of self-injection presents additional challenges, including the source of resupply and correct disposal of the device. DMPA-SC is still relatively scarce in Kinshasa (available in only 27.8% of pharmacies and health facilities) [3] and, even then, at prices that many women cannot afford. The major social marketing program provides DMPA-SC but uses its own personnel (“bees”) to administer it [21]. Will local health centers allow women to purchase doses of DMPA-SC for home use without charging them the client inscription fee? A Kinshasa-wide community-based program, AcQual III, launched in mid-2018 will include a monthly campaign day in every health zone, where nursing students and community health workers (nonmedical profile) will offer contraceptives at a highly subsidized price; as such, it could be a source of supply for self-injectors. Yet even then, will project personnel require demonstration that shows a woman to be competent in self-injection? The researchers on this study will address this set of operational challenges to the scale-up of DMPA-SC self-injection in the next phase of programmatic research in Kinshasa.

## References

[1] Ministère du Plan et Suivi de Mise en Oeuvre de Revolution de la Modernité (MPSMRM), Ministère de la Santé Publique (MSP), ICF International (2014). Deuxième Enquête Démographique et de Santé en République Démocratique du Congo 2013–2014.

[2] Muanda M., Gahungu Ndongo P., Taub L.D., Bertrand J.T. (2016). Barriers to modern contraceptive use in Kinshasa, DRC. PLoS One.

[3] Monitoring P. (2017). Accountability 2020 (PMA2020) Project, Kinshasa School of Public Health, Tulane School of Public Health and Tropical Medicine. https://www.pma2020.org/sites/default/files/MHM_Brief_Kinshasa_051018-Eng.pdf.

[4] Mukaba T., Binanga A., Fohl S., Bertrand J.T. (2015). Family planning policy environment in the Democratic Republic of the Congo: levers of positive change and prospects for sustainability. Glob Health Sci Pract.

[5] Programme National de la Santé de la Reproduction Planification Familiale: Plan National Stratégique à Vision Multisectoriel 2014–2020. Kinshasa 2014. http://familyplanning-drc.net/docs/Final%20Plan%20Strategique%20version%20officielle.pdf.

[6] Spieler J. (2014). Sayana® Press: can it be a “game changer” for reducing unmet need for family planning?. Contraception.

[7] Burke H.M., Mueller M.P., Perry B., Packer C., Bufumbo L., Mbengue D. (2014). Observational study of the acceptability of Sayana® Press among intramuscular DMPA users in Uganda and Senegal. Contraception.

[8] Khan S., Grady B., Tifft S. (2015). Estimating demand for a new contraceptive method: projections for the introduction of Sayana Press. Int J Gynecol Obstet.

[9] Keith B., Wood S., Tifft S., Hutchings J. (2014). Home-based administration of Sayana® Press: review and assessment of needs in low-resource settings. Contraception.

[10] Cameron S. (2013). Subcutaneous depo-medroxyprogesterone acetate. J Fam Plann Reprod Health Care.

[11] Burke H.M., Mueller M.P., Packer C., Perry B., Bufumbo L., Mbengue D. (2014). Provider acceptability of Sayana® Press: results from community health workers and clinic-based providers in Uganda and Senegal. Contraception.

[12] Cover J., Blanton E., Ndiaye D., Walugembe F., LaMontagne D.S. (2014). Operational assessments of Sayana® Press provision in Senegal and Uganda. Contraception.

[13] Polis C.B., Nakigozi G.F., Nakawooya H., Mondo G., Makumbi F., Gray R.H. (2014). Preference for Sayana® Press versus intramuscular Depo-Provera among HIV-positive women in Rakai, Uganda: a randomized crossover trial. Contraception.

[14] Prabhakaran S., Sweet A. (2012). Self-administration of subcutaneous depot medroxyprogesterone acetate for contraception: feasibility and acceptability. Contraception.

[15] Binanga A., Bertrand J.T. (2016). Pilot research as advocacy: the case of Sayana Press in Kinshasa, Democratic Republic of the Congo. Glob HealthSci Pract.

[16] Bertrand J.T., Makani P.B., Hernandez J., Akilimali P., Mukengeshayi B., Babazadeh S. (2017). Acceptability of the community-level provision of Sayana® Press by medical and nursing students in Kinshasa, Democratic Republic of the Congo. Contraception.

[17] Stout A., Wood S., Barigye G., Kaboré A., Siddo D., Ndione I. (2018). Expanding access to injectable contraception: results from pilot introduction of subcutaneous depot medroxyprogesterone acetate (DMPA-SC) in 4 African countries. Glob Health Sci Pract.

[18] Cover J., Namagembe A., Tumusiime J., Nsangi D., Lim J., Nakiganda-Busiku D. (2018). Continuation of injectable contraception when self-injected vs. administered by a facility-based health worker: a non-randomized, prospective cohort study in Uganda. Contraception.

[19] Burke H.M., Chen M., Buluzi M., Fuchs R., Wevill S., Venkatasubramanian L. (2018). Effect of self-administration versus provider-administered injection of subcutaneous depot medroxyprogesterone acetate on continuation rates in Malawi: a randomised controlled trial. Lancet Glob Health.

[20] Cover J., Namagembe A., Tumusiime J., Lim J., Drake J.K., Mbonye A.K. (2017). A prospective cohort study of the feasibility and acceptability of depot medroxyprogesterone acetate administered subcutaneously through self-injection. Contraception.

[21] Kwete D., Binanga A., Mukaba T., Nemuandjare T., Mbadu M.F., Kyungu M.T. (2018). Family planning in the Democratic Republic of the Congo: encouraging momentum, formidable challenges. Glob Health Sci Pract.

[22] Programme National de Santé de la Reproduction (2018). Offre communautaire de Sayana Press et Implanon NXT en République Démocratique du Congo: Résultats des Trois Etudes Pilotes.

